# Nanomaterials-Based Electrochemical Aptasensors for Rapid Detection of Pathogens and By-Products

**DOI:** 10.3390/molecules31040664

**Published:** 2026-02-14

**Authors:** Zhang Lei, Norjihada Izzah Ismail

**Affiliations:** 1 Bioinspired Device and Tissue Engineering (BIOINSPIRA) Research Group, Department of Biomedical Engineering and Health Sciences, Faculty of Electrical Engineering, Universiti Teknologi Malaysia, Johor Bahru 81310, Johor, Malaysia; zhanglei@graduate.utm.my; 2 Medical Devices and Technology Centre, Institute of Human Centered Engineering, Universiti Teknologi Malaysia, Johor Bahru 81310, Johor, Malaysia

**Keywords:** electrochemical aptasensor, aptamer, rapid detection, nanomaterials, pathogen, toxin, bacteria, virus

## Abstract

The rapid and accurate detection of pathogenic bacteria and viruses is essential for controlling infectious disease outbreaks and ensuring food safety. Conventional detection methods such as microbial culture, immunoassays, and polymerase chain reaction (PCR), although effective, often suffer from drawbacks including time-consuming procedures, complex operations, and limited multiplexing capabilities. In recent years, electrochemical aptasensors have emerged as a promising alternative for rapid detection of pathogenic bacteria, viruses, and by-products (toxins) due to their high sensitivity, excellent specificity, low cost, and potential for miniaturization. Aptamers can be applied as biorecognition elements of the biosensor, remarkably offering advantages such as high binding affinity, thermal stability, and ease of chemical synthesis. Meanwhile, nanomaterials which provide large surface area, superior conductivity, and modifiable surfaces are widely employed in signal amplification and sensor platform construction. This review discusses the cutting-edge innovations in electrochemical aptasensors in recent years that utilize various types of nanomaterials to accurately identify and quantify diverse types of pathogens and toxins. This review focuses on nanomaterials such as metal nanostructures, carbon nanomaterials, metal, metal oxides, and carbon nanocomposites that can synergistically enhance detection sensitivity, specificity, and operational stability. This review also highlights the promising practical application of the proposed electrochemical aptasensors in clinical diagnostics, environmental monitoring, and food safety.

## 1. Introduction

Pathogens such as bacteria and viruses spread at extremely fast speeds, posing unprecedented challenges to global public health and cross-border epidemic prevention and control [1,2]. Specifically, bacteria are one of the main sources of epidemics and are mainly found in contaminated water, food, and other biological samples, making them contagious [3,4]. History has also shown that viruses can easily acquire the ability to initiate and spread a pandemic due to their rapid spread and high rate of transmission of new mutations, as well as the difficulty faced by humans in obtaining rapid and accurate diagnostics, specific vaccines, or therapeutic drugs in a short period of time [5,6].

While rapid, sensitive, and specific detections of pathogenic bacteria and viruses are still challenging, techniques involving cell culture, immunological reaction, polymerase chain reaction (PCR) and mass spectrometry are the current mainstream detection methods [7,8,9,10,11,12]. Since cell culture-based methods include selective enrichment, biochemical screening, and humoral confirmation steps, it takes 24–48 h to obtain preliminary results of identification, which is time-consuming and labor-intensive [7,13,14]. Immunoassays such as enzyme-linked immunosorbent assay (ELISA) have been widely used for pathogen detection. ELISA has been proven to be the gold standard for detecting *Staphylococcus aureus* enterotoxins in food production facilities [8,10]. However, three key limitations restrict its application. The assay requires labor-intensive steps (e.g., repeated washing, incubation), which prolongs the processing time by 2–4 h compared to the automated methods. In addition, obtaining reliable results typically require 50–100 μL of sample, which is challenging in situations where sample scarcity exists (e.g., neonatal blood). ELISA has sensitivity limitations in which the detection thresholds are limited to the nanomolar range (≥1 nM), therefore preventing detection of early biomarkers such as interleukin-6 in sepsis (0.1–0.5 nM) [15,16,17].

PCR technology can improve sensitivity (attomole detection) and specificity, shortening the diagnostic cycle to 1–24 h. Multiplex platforms of PCR enable simultaneous detection of bacteria such as *S. aureus* and *Staphylococcus epidermidis* within 90 min [12]. Real-time reverse-transcriptase PCR (qRT-PCR) is able to detect 14 respiratory viruses including SARS-CoV-2 and influenza A virus with clinical consistency up to 95% [18]. Despite these advantages, several disadvantages remain. In terms of pretreatment, a 12–24 h pre-enrichment step is usually required to amplify bacterial DNA and to exclude inactive pathogens [19]. The other issue is linked to matrix interference. Complex samples (e.g., meat homogenates) normally contain PCR inhibitors (e.g., polysaccharides, fats) which can reduce the amplification efficiency by 30–60% unless the samples are pretreated by centrifugation or filtration [9,20].

Biosensors have been introduced as another strategy for pathogen detection and are notably preferred due to several advantages of these instruments: highly sensitive and selective, rapid, easy sample preparation, are easy to use, allow on-site detection and are cost-effective [21,22]. The biosensor architecture integrates three core modules: (1) biorecognition (e.g., engineered antibodies/aptamers), (2) transducer (e.g., nanomaterial-enhanced electrodes), and (3) signal processor (i.e., microchip-embedded algorithms) to achieve automated quantitative analysis of pathogens [23]. Biorecognition is the specific interaction of antibodies or aptamers with target analytes, which improves the sensitivity and selectivity of biosensors, achieves high-precision detection, and minimizes other interferences in the sample [21].

In the past 10 years, aptamer has gained enormous attention for use in biosensor development. A study revealed that the carbon nanotube (CNT)-based field effect transistor (FET) biosensor achieved highly sensitive detection of foodborne pathogens *Salmonella* enterica and *S. aureus* through aptamer functionalization. The aptamer biosensor (aptasensor) maintained a stable performance in six complex food matrices, with a detection limit as low as 3.1 CFU, a response time as short as 200 s, and no cross-reaction with closely related bacteria such as *Listeria* [24]. Another study developed an electrochemical aptasensor for the detection of *Acinetobacter baumannii* with a detection limit of 1 CFU/mL. The functional groups, namely, hydroxyl and carboxyl groups in the synthetic carbon quantum dots (CQD), reduced graphene oxide (rGO), multi-walled carbon nanotubes (MWCNTs), and chitosan (CS) nanocomposite help immobilize a large number of aptamers onto the electrode which effectively enhanced the stability and activity of the aptasensor. This nanocomposite material exhibited high conductivity and fast electron-transfer kinetics which promote enhanced sensitivity and specificity. In addition, the large surface area offered by the hemin-graphite oxide (H-GO) attached to the secondary aptamers increased the aptamer surface density, leading to signal amplification and better sensitivity. This aptasensor was used to monitor the presence of live *A. baumannii* cells in human serum and skimmed milk powder, working as a platform for early detection and diagnosis of this pathogen-associated infection [25].

## 2. Electrochemical Biosensors and Aptamers for Bacterial and Viral Detection

Electrochemical biosensors convert biochemical signals into electrical signals through electrodes and provide output in qualitative and quantitative information of target analytes after processing [26]. Compared with traditional methods, the advantage of electrochemical biosensors is that they can immobilize aptamers to form highly sensitive electrochemical aptasensors. Aptamers can be fixed to the surface of gold or carbon-based electrodes through chemical bonds. When combined with nanostructured amplification strategies, these platforms achieve single-cell-level detection in bacterial detection and exhibit picomolar to femtomolar limits of detection in viral detection while maintaining specificity in complex sample media such as food, water, and serum [27].

Aptamers are synthetic single-stranded oligonucleotides (DNA or RNA) developed through the systematic evolution of ligands by exponential enrichment (SELEX) process and are commonly 25–80 nucleotides in length [20,27,28]. Aptamers can fold into a defined three-dimensional structure following intramolecular base-pairing which results in their high affinity and specificity to bind with targets such as small molecules, toxins, bacteria, and viruses [27,28,29]. The aptamers can fold into hairpin, loops, stem, bulges, and G-quadruplexes for molecular recognition and non-covalent interactions such as hydrogen bonding, polar contacts, van der Waals forces, hydrophobic interactions, stacking of aromatic rings and electrostatic interactions mediate the aptamer–target binding [28]. Since their first report in 1990, aptamers have been widely studied due to their superior properties compared to antibodies including non-immunogenicity, high specificity, easy modification, stability, shorter production time, lower cost, and scalable synthesis [29,30]. Interestingly, aptamers have been shown to be capable of differentiating thousands of proteins and nucleotides within a short time as well as identifying small variations between proteins with similar structures [29].

SELEX is an aptamer screening strategy which employs a series of selection and amplification steps. It starts with screening a random library of single-stranded DNA or RNA against the specific target under specific conditions such as a specific temperature and salt concentration. The nucleic acid molecules bound with the target of interest are isolated from the unbound targets, and PCR amplification is used to selectively enrich these target-specific aptamers, followed by high-throughput sequencing [28,30]. Isolation of aptamers that bind specifically to the target may require 1 to 20 rounds of SELEX depending on the nature of target [28]. While the SELEX process has been time-consuming in the past decade, improvements in SELEX technology through in silico pre-screening have shortened the aptamer development process and reduced the production costs [30,31,32,33]. With high-efficiency SELEX technology, pathogen-specific aptamers can be rapidly obtained during new epidemic outbreaks, such as the recent acquisition of specific aptamers for severe acute respiratory syndrome coronavirus 2 (SARS-CoV-2) [34,35,36].

Aptamers can identify pathogens by targeting surface antigens such as lipopolysaccharide or unknown structures. Table 1 lists several aptamers and their targets. At present, numerous bacterial aptamers are available, primarily target pathogens such as *S. aureus*, *Listeria monocytogenes*, *Salmonella* spp., *Escherichia coli*, *Streptococcus pyogenes*, *Mycobacterium tuberculosis*, and *Pseudomonas aeruginosa*. The aptamers of various lengths have been used [33,37,38,39,40,41,42,43,44,45]. It was also highlighted in the past study that the MPT64 aptamer offers more sustainable, stable, and low-cost alternatives to antibodies in the development of point-of-care biosensors, allowing shorter detection time of 30 min. In addition, MPT64-specific aptamers enable sensitive electrochemical impedance detection of secretory antigens of *M. tuberculosis* at a limit of detection of 81 pM without the need for fluorescent or enzyme labeling [38].

Viral pathogens including human immunodeficiency virus (HIV-1), hepatitis B virus (HBV), and SARS-CoV-2 are increasingly being targeted by aptamer-based detection platforms. Cossettini and colleagues [33] has computationally designed 18 high-affinity aptamers (Kd < 1.2 nM) for the SARS-CoV-2 spike receptor binding domain using a deep learning-accelerated SELEX workflow (AptaNet v2.0) and achieved high specificity in distinguishing Omicron subtypes (BA.5 vs. XBB.1.5) using electrochemical impedance spectroscopy. For detection of glycoprotein (GP) and soluble glycoprotein of Ebola virus (EBOV sGP) (Table 1), a past study revealed that all three studied aptamers, namely, 6011, 6012, and 39SGP1A, exhibited similar affinities to sGP and GP1,2 proteins. One of the aptamers (6011) was selected as an electrochemical sensor element and the study confirmed that Ebola GP1,2 proteins on pseudotyped virions can be detected while high sensitivity of EBOV sGP detection was observed with limit of detection of 150 pM [39].

Pathogenic bacteria such as *Salmonella enterica* serovar *Typhi* (typhoid fever), *E. coli* O157:H7 (hemolytic uremic syndrome), *S. aureus* (toxic shock syndrome), and *Shigella dysenteriae* (bacillary dysentery) cause more than 1.5 million deaths worldwide each year, mainly in resource-poor settings [49,50,51,52,53]. These pathogens employ different virulence mechanisms. *Salmonella enterica* serovar *Typhi* invades intestinal epithelial cells via a type III secretion system, causing systemic infection with a mortality rate of 10–20% if untreated [50]. *E. coli* O157:H7 produces Shiga toxin, which damages renal endothelial cells and may lead to acute kidney injury (AKI) progression to renal failure in pediatric cases [51]. *S. aureus* secretes various virulence factors including superantigens (e.g., toxic shock syndrome toxin-1 [TSST-1]) and causes bacteremia which results in shock or sepsis with mortality rates between 10 and 80% [52]. *Shigella* species, which is responsible for up to 165 million infections each year, colonizes the colonic mucosa using actin-based peristalsis and can cause severe bloody diarrhea [53].

Deadly viruses such as influenza viruses, Ebola virus, human immunodeficiency virus (HIV), Hantavirus and Dengue virus can affect people in a specific region or from all over the world and are therefore accounted as significant threats to human health [26,54,55,56,57,58]. SARS-CoV-2, the pathogen of COVID-19, caused nearly 14.83 million excess deaths globally as estimated by WHO [59]. COVID-19 has been estimated to have caused global economic losses between US $77 billion and US $2.7 trillion in 2019, and since then has increased the burden on medical infrastructure worldwide due to the elevated medical costs, healthcare use, and medications [60,61]. It is worth noting that bacterial coinfection and secondary bacterial infection are also considered as the key risk factors for the observed severity and mortality of COVID-19 patients [62,63,64]. The viral infection weakens the host immune system, paving a way for the development of viral–bacterial coinfections [65,66].

Most importantly, the re-emergence of viral diseases poses a continuous threat to humans and healthcare settings. Recent reports revealed that over 3000 cases of monkeypox, a rare zoonotic viral disease transmitted from animals to humans, have been recorded in more than 50 countries since early May 2022 [67]. Marburg virus (MARV) is a highly pathogenic virus that causes a filoviral hemorrhagic fever characterized by hemorrhagic manifestations and multi-organ failure, with a mortality rate of up to 88% among confirmed cases in the 2022 Marburg outbreak in Equatorial Guinea [6,68,69]. Multiple outbreaks of MVD have occurred in Africa over the past fifty years, and two outbreaks were also reported in Europe [70]. Since its discovery near the Ebola River in 1976, more than 30 discrete outbreaks of Ebola virus disease (EVD) have been documented, with clusters occurring in Central and West Africa. In 2021, Guinea experienced a new EVD outbreak [71]. The 2014–2016 West African epidemic with 28,646 cases and 11,323 deaths, and 2018–2020 Kivu outbreak with 3470 cases and 2287 deaths generated over 17,000 survivors with persistent post-Ebola sequelae (ocular/neurological complications) [57,72].

Oncogenic viruses cause 15–20% of cancer incidence worldwide; for example, human papillomavirus (HPV) causes hundreds of thousands of cases of cervical cancer each year [73,74], while HBV and hepatitis C virus (HCV) work together to cause 80% of hepatocellular carcinoma in endemic areas, and this proportion is even higher in high-endemic areas such as sub-Saharan Africa and East Asia [75]. Cancer is a significant threat to human health, causing nearly 10 million deaths globally every year, and viral infections are responsible for a considerable proportion of these deaths [76]. Therefore, the use of electrochemical aptamer sensors to continuously monitor the presence and quantity of tumor viruses has important clinical significance for early cancer diagnosis and treatment [77].

## 3. Nanomaterials in Electrochemical Aptasensors for Detection of Pathogens and By-Products

**Figure 1:**
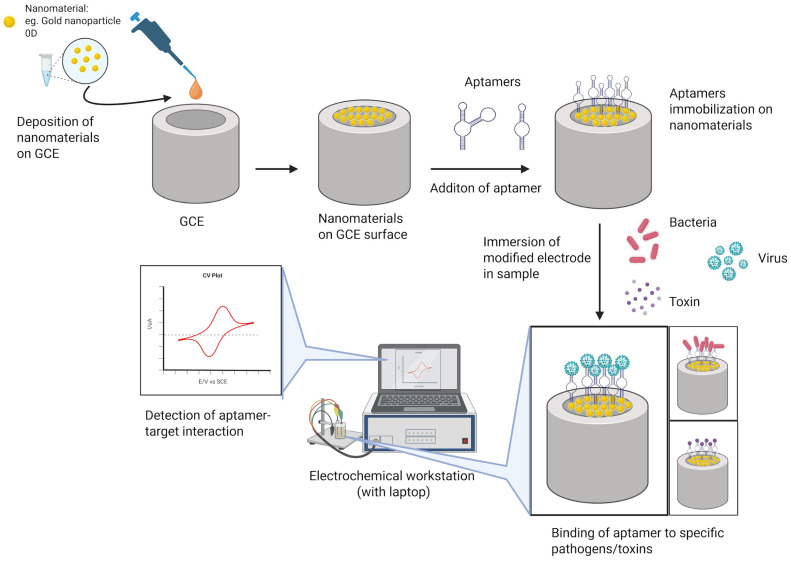
Modification of electrochemical sensor with nanomaterials and aptamers for pathogens and toxins detection.

Electrochemical aptasensors have gained prominence for pathogen detection, considering the critical needs of rapid detection in clinical and food safety contexts [3,78]. These sensors commonly utilize nanomaterials as dual-functional platforms: immobilizing aptamers and amplifying electrochemical signals. Nanomaterials, owing to their small particle size in the nanoscale range (1–100 nm), have been utilized in development of numerous biosensing tools for a wide range of applications such as biotechnology, medical diagnostic, environment, agriculture, and food safety [79]. Figure 1 depicts the utilization of nanomaterials in the electrochemical aptasensor where the nanomaterials are deposited onto a working electrode such as a glassy carbon electrode (GCE).

Five nanomaterial classes dominate current research: metal/metal oxide nanoparticles (e.g., gold (Au), silver (Ag), magnetite (Fe_3_O_4_)), carbon-based materials (e.g., graphene, carbon nanotubes), quantum dots (QD), polymer nanoparticles, and hybrid composites [37,78,80]. The performance superiority of the nanomaterials-based electrochemical aptasensors arises from the nanomaterials contributions such as enhanced surface area, increased sensitivity and specificity, multiplexing, multi-analyte detection, ease of functionalization, rapid detection, real-time monitoring, reduce false positives and negatives, and consistent performance [81,82]. Interestingly, nanomaterials can be integrated into the working electrode of electrochemical sensors via various techniques including electrodeposition, surface modification, layer-by-layer assembly, sol–gel technique, and printing technologies [82].

**Figure 2:**
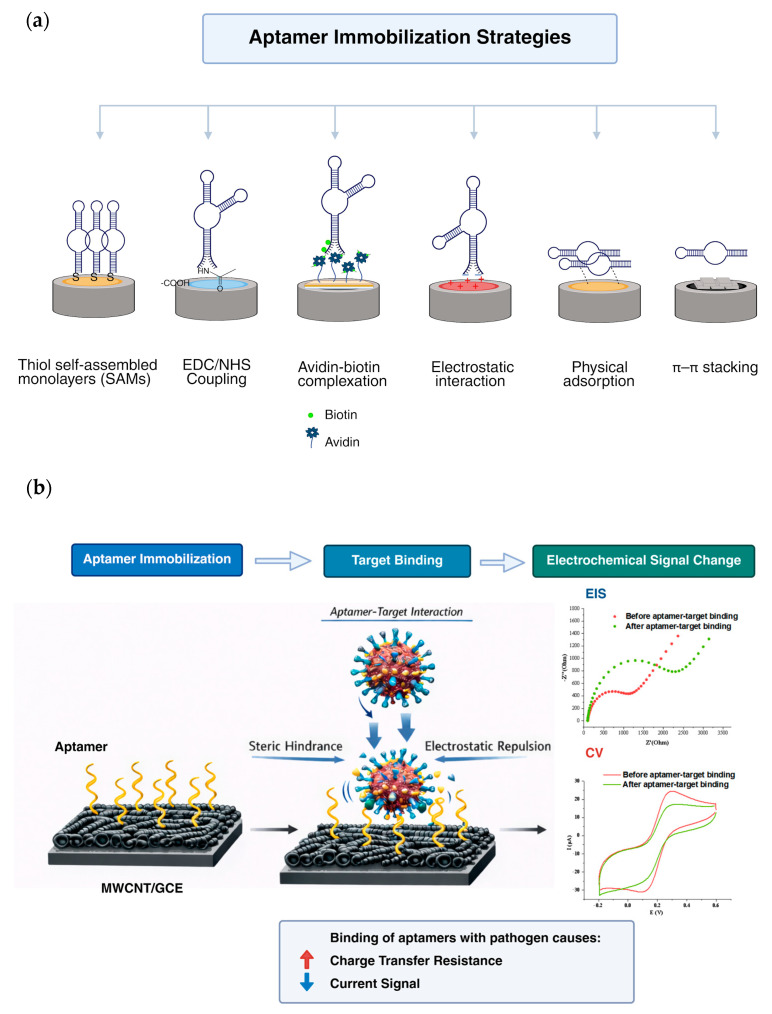
Schematic illustration of (**a**) strategies of aptamers immobilization on nanomaterials for electrochemical aptasensors and (**b**) binding of aptamers with the target to generate electrical signals.

As the bioreceptor of aptasensors with stable secondary structure, aptamers are well-known for their high binding affinity and selectivity for specific target molecules, such as amino acids, proteins, enzymes, and metal ions [79,81]. These properties are attributed to the ability of the aptamers to fold via electrostatic interactions, van der Waals forces and hydrogen bonds, forming thermodynamically stable three-dimensional structures. The accuracy and sensitivity of the assays can be further improved while the limit of detection can be reduced by utilizing the combination of aptamers with different nanomaterials [79]. To ensure this, aptamer conjugation with nanomaterials is a crucial step for effective functionality of aptasensors especially to preserve the short single-stranded DNA (ssDNA) or RNA biophysical characteristics and binding abilities. Several methods are available for the conjugation, namely, physical adsorption, covalent and non-covalent attachments [81]. Figure 2a presents the examples of aptamer immobilization strategies on nanomaterials for electrochemical aptasensors. After successful aptamer immobilization on the nanomaterials, subsequent binding between the aptamer and target will produce a measurable electrical signal, and alteration in electrical signals before and after binding can also be observed (Figure 2b).

### 3.1. Metal Nanoparticles

Metallic nanostructures are becoming the key components in the optimization of aptasensors due to their high surface-area-to-volume ratio, fast electron transfer kinetics, increased loading capacity, mass transport of reactants, and inherent cytocompatibility [79,83]. Gold (Au) and silver (Ag) nanoparticles are widely used in customized sensor design due to their excellent conductivity, and controllable size and morphology (e.g., nanospheres, nanorods, nanocages, nanowires, etc.) [79,84]. Gold nanoparticles (AuNPs) have gained worldwide attention for diverse diagnostic and therapeutic applications due to their inertness, biocompatibility, low toxicity, chemically stable, localized surface plasmon resonance (LSPR) properties and easy modification [79,85]. The conventional synthesis method of AuNPs is via reduction of gold (III) derivatives using citrate or other reducing agents such as gallic acid [86].

Similarly, silver nanoparticles (AgNPs), which can be synthesized conventionally through chemical and physical methods, are cost-effective and have demonstrated minimal cytotoxicity and immunological response. As one of the preferred nanomaterials for electrochemical sensors, AgNPs enable rapid and sensitive detection, which supports its utilization in point-of-care devices [87]. In the past years, plant extracts and microbial (i.e., bacteria, fungi, yeasts, actinomycetes, and viruses) green synthesis methods of AuNPs and AgNPs have been developed, which was proven effective for the development of sustainable production systems [85,86,87,88]. These environmentally friendly approaches offer several advantages including the absence of toxic by-products from the chemical-reducing agents which could be introduced to the environment and pose health hazards, reduced energy demands and costs for nanoparticle production, and enhanced scalability by using non-toxic materials [86,87].

Many studies have reported successful detection of pathogenic bacteria and viruses using AuNPs-modified electrochemical aptasensors [89,90,91,92,93,94,95,96]. Zarei et al. [90] have successfully applied a strategy of thiol-bonded aptamer fixation for the detection of *S. dysenteriae*. The aptasensor utilized a glassy carbon electrode (GCE) modified with AuNPs through electrodeposition technique and the AuNP/GCE was combined with a thiolated aptamer via self-assembly immobilization to detect *S. dysenteriae* in milk and water samples. This study reported that the aptasensor has a detection limit of 100 CFU/mL (Table 2) and a recovery rate of 93.26–132.95%. The aptasensor also showed higher selectivity, in which the presence of other bacteria and dead *S. dysenteriae* did not affect its performance. Another past study revealed that metallic nanoparticles such as AuNPs can be coupled with rolling circle amplification (RCA) technology to further increase sensor sensitivity for detection of live *S. typhimurium*. The RCA products which are the massive, long DNA molecules with multiple tandem-repeat sequences can later hybridized with the detection probe on the aptasensor surface [91].

Different types and/or functionalization have also been performed on AuNPs for its utilization in electrochemical aptasensor. Porous AuNPs (pAuNPs) which were synthesized by reducing the agent-assisted excessive galvanic replacement method with diameter distribution of 60–110 nm has been utilized in the fabrication of multifunctional DNA structure on pAuNPs/Au electrode for detection of hemagglutinin (HA) protein, an envelope protein of avian influenza virus (H5N1) [92]. From the cyclic voltammogram (CV) results, the authors pointed out that binding of HA protein to the bioprobe AIapt/Zyme/SH-DNA 3WJ hampered electron transfer, resulting in increasing redox peaks of AIapt/Zyme/SH-DNA 3WJ with decreasing HA protein concentration. That study also suggested that the electrode modified with pAuNPs achieve higher coverage and thus enhanced signal due to the higher surface roughness and active area the pAuNPs provide [92].

A past study revealed that their aptasensor composed of indium tin oxide (ITO) glass electrode modified with AuNPs–Cysteine–aptamer exhibited increased current intensity with an increase in SARS-CoV-2 virus spike (S) protein concentration between 10 pM and 6 nM as detected by differential pulse voltammetry technique (DPV) [93]. Roushani et al. [96] modified GCE with electrodeposited AgNPs and immobilized aptamers via covalent bonding between the aptamer amino group and AgNPs. The study successfully detected *P. aeruginosa* at the concentrations of 10^2^–10^7^ CFU/mL with a limit of detection of 33 CFU/mL (Table 2). The study highlighted that the presence of AgNPs on the electrode surface improved the signal due to its large surface area and fast electron transfer. The study also reported that 50 min would be suitable for target binding time while 4 h was required to allow optimal aptamer immobilization [96].

Several strategies have been implemented to further improve the performance of electrochemical aptasensors. For instance, nonspecific adsorption in complex samples and electrode biofouling can reduce sensitivity and specific bacterial detection, and these problems can be alleviated using polymers such as polyethylene glycol (PEG) as an antifouling agent [100]. In addition, dual aptamer synergistic recognition based on aptamers that target specific proteins can further optimize the sensitivity of the electrochemical aptasensors. For example, utilization of different aptamers that target the same MPT64 protein, which is the protein secreted in the early and middle period of *M. tuberculosis* growth, was able to further improve the sensitivity of the aptasensor supported by a low detection limit of 10 fg/mL [101]. Multiple aptamers have also been used in the development of multiplexed electrochemical aptasensors, mainly to allow simultaneous determination of substances such as five harmful marine toxins in tap water [95].

### 3.2. Metal/Metal Oxide Nanocomposites

Metal/metal oxide nanocomposites have been utilized to modify the working electrode in electrochemical aptasensor research studies. Nanocomposites are hybrid materials that can be composed of metallic, non-metallic, and polymeric materials. Incorporation of carbon nanotubes to metal nanoparticles, for example, CNT and AuNP, form AuNP–CNT nanocomposites which display enhanced performance and properties. The AuNP–CNT nanocomposites could provide exceptional electrical conductivity, high sensitivity and selectivity, and easy surface modification, which favors their use in sensor application [102]. Many research studies have utilized metal nanocomposites for development of electrochemical aptasensors [11,103,104,105,106,107,108,109,110,111]. The AuNPs/SPANI nanocomposite aptasensor developed by Gao et al. [103] used the anti-biological contamination properties of sulfonated polyaniline (SPANI) to act as an antifouling agent and combined it with the signal amplification effect of AuNPs to directly detect *S. aureus* with detection limit of 2 CFU/mL (Table 3).

Ranjbar and Shahrokhian [104] fabricated an electrochemical aptasensor using gold nanoparticles/carbon nanoparticles/cellulose nanofibers nanocomposite (AuNPs/CNPs/CNFs) for sensitive and selective detection of *S. aureus*. The nanocomposites exhibited excellent conductivity, good biocompatibility, and high surface area, which contributed to high sensitivity with a limit of detection of 1 CFU/mL for the aptasensor and precise detection of *S. aureus* in the spiked human serum (Table 3). Hydroxyapatite (Hap) has been used in combination with Ag, zinc oxide (ZnO) and conductive polymer polythiophene (PP) for detection of *H. pylori* heat shock protein (HSP 60) [107]. They pointed out that the multiple functional group sites of Hap-Ag-ZnO-PP composites facilitated the aptamer immobilization through the aptamer’s NH_2_ group. A prominent decrease in current and increase in resistance was evident following hybridization of aptamer and Hsp 60, indicating successful attachment of Hsp 60 with the aptamer that formed a barrier layer which blocks the transfer of electrons. Their proposed aptasensors showed high sensitivity with detection limit of 0.429 nM (Table 3).

A 3D electrochemical aptasensor containing phosphorene–gold nanocomposites (BP-AuNCs) was developed by Jiang et al. [109] for detection of norovirus (Table 3). They fabricated the aptasensors using pins and fabric cloth which are low cost, and the incorporation of multiple layers of BP-AuNCs has resulted in ultrasensitive detection of the recombinant norovirus-like particles (VLP) with a limit of detection of 0.28 ng/mL. The sensitivity of the aptasensor could be attributed to the BP-AuNCs which provide a durable 3D support structure for the target-specific aptamer immobilization while enhancing the electron transfer process at the interface. The study also pointed out that the aptasensor demonstrated high specificity for norovirus; current responses of interfering astrovirus and rotavirus were close to blank while the current responses of the virus mixture was comparable to that of only norovirus. It was observed that the recovery from the oyster spiked samples was 97–106%, supporting its potential use for detection in food samples [109].

Metal oxide nanoparticles such as cerium oxide (CeO_2_), zinc oxide (ZnO), iron oxide (Fe_3_O_4_), manganese dioxide (MnO_2_) and titanium dioxide (TiO_2_) are attractive fundamental building blocks for advanced electrochemical aptasensor architectures. Notably, metal oxide NPs have a simple preparation process, easy size, shape and porosity modification, high stability, no swelling variations, easy functionalization which is made possible due to the negative surface charge, and easy incorporation in both hydrophilic and hydrophobic systems [112]. On top of that, some metal oxide NPs such as MnO_2_ and Fe_3_O_4_ are favored for diagnostic purposes due to their non-toxicity and strong peroxidase-like activity, as well as low production cost as compared to natural enzymes production [113]. Specifically, nanomaterials with intrinsic enzyme-like activities are known as nanozymes. Nanozymes encompass a large number of nanomaterials which can be synthesized and display excellent catalytic activities as they mimic natural enzymes’ structures and functions (e.g., hydrolase, peroxidase, catalase, oxidase, etc.) [114].

Sharma et al. [115] synthesized ZnO nanorods by thermal method while AuNPs and AgNPs were prepared chemically. The Ag-Au-ZnO/origami-based electrochemical paper-based analytical device (oPAD) was used to fix the CHIKV aptamer onto it, forming an aptamer/Ag-Au-ZnO/oPAD. This aptasensor has shown ultrasensitive detection of Chikungunya virus antigen (CHIKV-Ag) with a linear range of 1 ng/mL–10 µg/mL (Table 4), and a detection limit of 1 ng/mL using CV. From the field emission scanning electron microscopy (FESEM) images, the spherical Au-Ag NPs were seen clustering on the surface of ZnO nanorods, which favorably contribute to a high sensitivity of 1.36 μA μgmL^−1^cm^−2^ as observed for this aptasensor. This improved sensitivity could be attributed to the enhanced charge-transfer properties provided by the tertiary nanocomposite. The aptasensor exhibited rapid detection, high stability, high sensitivity to serum sample, good reproducibility, and provided confidentiality in the form of a closed environment that protects electrodes from dust and external elements [115].

Another past study by El-Wekil and colleagues [116] developed an aptasensor by immobilizing aptamer on gold nanoparticles modified magnetic nanoparticles (apt-AuNPs@Fe_3_O_4_) (Table 4). In the presence of *S. aureus* as the target bacteria, a molecular imprinted polymer (MIP) film using o-phenylenediamine was electro-polymerized on the surface of the as-synthesized nanocomposite to fabricate a MIP-apt-AuNPs@Fe_3_O_4_/GCE. The *S. aureus* was later removed to allow binding of the *S. aureus* in complex matrices on the aptasensor at the imprinted sites. Their study revealed that the MIP-aptasensor demonstrated an ultrasensitive detection with a low limit of detection of 1 CFU/mL and a wide linear range of 10^1^–10^7^ CFU/mL. The specificity studies using CV were conducted in the presence of organic compounds such as urea, glucose, ascorbic acid, uric acid, methionine, glycine, alanine, lysine, arginine, and lactic acid (300 µM each), as well as other bacteria and *C. albicans* at a concentration of 10^6^ CFU/mL. They found out that reduced current was only observed in the presence of *S. aureus*, indicating the good interaction of aptamer and well-fitted MIP spaces for the *S. aureus* [116].

### 3.3. Carbon Nanomaterials and Nanocomposites

Carbon-based nanomaterials, which include carbon nanotubes (CNTs), graphene, graphene oxide, nanographite, fullerene, etc., are widely used as the core materials of electrochemical aptasensors. Their unique properties including high electron mobility, good electrical conductivity, large specific surface area, high stability, biocompatibility, hydrophilicity, and flexible interface functionalization capabilities have attracted their use for various applications such as in the field of medicine, electronics, environmental monitoring and food safety [29,79,81]. CNTs are composed of single-walled carbon nanotubes (SWCNTs) and multi-walled carbon nanotubes (MWCNTs). The SWCNT is a single tube with a common diameter of 0.4–2 nm. The MWCNTs which consist of several concentric tubes normally are having a diameter of 2–100 nm and the sizes are directly influenced by their synthesis method [119]. CNTs are known for their exceptional mechanical, electrical, and thermal properties, rendering them a suitable platform for the transducer component in aptasensors. The tensile strengths exceeding 100 GPa allow CNTs to withstand mechanical stresses and harsh environmental conditions while their good electrical conductivity ranging from 10^3^ to 10^4^ S/cm facilitate sensitive and rapid signal transduction [81].

Graphene can easily be functionalized and exhibits excellent electrical, electrochemical, and physicochemical properties. It is also highly sensitive to external stimulus and permits ssDNA binding through non-covalent π–π interaction or electrostatic interaction with DNA bases which promotes biomolecule immobilization [120]. Graphene oxide (GO), a derivative of graphene, is rich in oxygen functional groups such as hydroxyl and carboxyl groups [83]. Both graphene and GO demonstrate excellent optoelectronic properties and can detect biomolecules with high sensitivity and selectivity [79,104]. GO is commonly transformed to reduced graphene oxide (rGO) for use in sensor application, where the conversion can be achieved by the hydrothermal and chemical reduction of GO [83]. The higher hydrophilicity, higher current density, superior electrocatalytic property, and easier functional groups attachments, such as those for aptamer immobilization or for binding to a transducer or support surface, are the among the advantages offered by rGO [83,119]. However, there are some limitations of rGO where it is prone to aggregating or possibly restacking to graphite following van der Waals forces and intense layering, which may limit its application in electrochemical sensing due to a reduction in total surface area [83].

Research studies have been conducted using carbon nanomaterials and aptamers for electrochemical detection of pathogens. Kaur et al. [121] constructed a hierarchical nanostructure of 3D bridged rebar graphene (BRG) using MWCNTs which was modified by the chemical facilitated unscrolling method, followed by bridging with terephthalaldehyde (TPA). The BRG showed enhanced electrical properties supported by the eleven-fold increase in the current and facile chemical functionality and was thus functionalized with polyL-lysine (PLL) and aptamer, forming an aptamer-functionalized BRG (BRG/PLL/Apta) for detection of *E. coli* O78:K80:H11. The work has successfully achieved ultrasensitive detection of *E. coli* O78:K80:H11 with a limit of detection of 10^1^ CFU/mL and detection range of 10^1^–10^6^ CFU/mL in water and other samples (Table 5). It was also pointed out that there was minimal interference in the presence of other bacterial species such as *L. monocytogenes*, *Bacillus subtilis*, *Proteus vulgaris*, *E. coli* DH5α, etc., indicating the high specificity of the electrochemical aptasensor. The interaction of aptamer and *E. coli* O78:K80:H11 led to high specificity of the aptasensor, and this result was supported by their scanning electron microscopy (SEM) and confocal microscopy images. The charge transfer resistance (Rct) values showed an increasing trend with increase in bacterial concentration in the range of 10^1^–10^6^ CFU/mL, highlighting successful *E. coli* O78:K80:H11 binding with aptamers on the sensor surface. The aptamer–target binding on the electrode surface prevented electron transfer from the redox probe which was attributed to the steric hindrance and insulating layer formed by the large bacterial cells.

Another study utilized an amino-modified aptasensor fabricated using MWCNTs deposited on ITO electrode (ssDNA/MWCNT/ITO) for detection of pathogenic *Salmonella* bacteria [122]. The MWCNTs were electrodeposited onto the ITO electrode at 30 V for 1 min followed by functionalization of MWCNT surface using EDC-NHS chemistry. The ssDNA/MWCNT/ITO was formed by dropping the amino-modified *Salmonella* DNA aptamer onto the activated electrode. Their study revealed that a stable and regular dispersion of MWCNTs on the ITO electrode was observed from the electrodeposition process. The high specific surface area of MWCNTs promoted aptamer immobilization by binding with the reactive carboxyl groups on the MWCNTs surface and the π-π stacking between MWCNTs layers and aptamer nucleotide bases allowed binding of the aptamer on the electrode. This study identified that 5 μmol/L was the optimum concentration of DNA aptamer needed for their aptasensor development. The signal amplification effects were contributed by the MWCNTs as these nanomaterials have excellent electrical conductivity and large specific surface area. Decreased peak currents and increased impedance were evident with increasing concentrations of *Salmonella* cells. The presence of high concentration of *Salmonella* enabled more aptamer–target binding on the aptasensor surface, which reduced the effective surface area and subsequently inhibited electron transfer. Additionally, this study also compared the sensitivity limit using the PCR technique, in which the PCR resulted in a limit of detection of 10^2^ CFU/mL for both *Salmonella enteritidis* and *S. typhimurium* while their aptasensor worked exclusively sensitive with a detection limit of 10^1^ CFU/mL (Table 5), suggesting its suitable application for food samples.

Carbon-based nanocomposites emerge as another pivotal material in electrochemical sensing. Pathania et al. [123] synthesized MoS_2_-rGO nanocomposites for the electrochemical aptasensor development which contributed to enhanced conductivity and easy biomolecular functionalization. The attachment of anti-Vi antigen aptamers on the MoS_2_-rGO nanocomposite through thiol linkage enables sensitive and specific detection of *S. Typhi*, differentiating it from other *Salmonella* serovars and enteric pathogens. The advantages of using MoS_2_ was related to the enhanced electrical, optical, and thermal properties as well as the tunable band gap. Their study integrated MoS_2_ with rGO to prevent restacking of MoS_2_ and improve the electrical conductivity. The aptasensor achieved a limit of detection of 100 pg/mL (Table 6) and high specificity was observed through selective binding of aptamer and Vi-expressing *S. Typhi* cells in the presence of different polysaccharides of other bacterial origin as supported by the confocal images. This study has successfully validated the aptasensor using urine and sera specimens spiked with Vi antigen. It was suggested that this aptasensor could be useful to track carriers of *S. Typhi* and evaluate disease prognosis, providing an easy-to-perform, rapid and reliable diagnostic test [123].

Appaturi et al. [128] hybridized rGO with carbon nanotubes (CNTs) for rapid detection of *S. typhimurium* (Table 6). The CNTs acted as one-dimensional spacers that inhibit the stacking of graphene sheets and provide large surface area, effectively increasing the loading capacity of the aptamers. The amino-modified aptamers bound covalently with the carboxyl groups of the rGO-CNT, forming amide bonds. The developed ssDNA/rGO-CNT/GCE aptasensor exhibited high sensitivity with a detection limit of 10^1^ CFU/mL. The high specificity of the aptasensor could be attributed to the intermolecular folding of the aptamers that recognize only the target bacteria and prevent binding with non-*Salmonella* bacteria. The rGO-CNT contributed to enhanced electrical properties and detection of whole bacterial cell without any pretreatment or DNA extraction steps, which was made possible through the immobilization of amino-modified DNA aptamer to the nanocomposites. Differential pulse voltammetry (DPV) results revealed a decrease in oxidation current density at the potential of 0.23 V upon binding of *S. typhimurium* onto the ssDNA/rGO-CNT/GCE in a concentration-dependent manner in comparison to the ssDNA/rGO-CNT/GCE which exhibited a prominent increase in oxidation. This observed decrease in current density could be related to the bacterial cell having negative charge on its membrane which interferes and blocks electron transfer to the electrode surface [128].

Several studies have proposed electrochemical aptasensors for rapid and sensitive detection of viruses including hepatitis B (HBV) and C (HCV) viruses, and human papilloma virus (HPV) [131,132,133,134]. In a study by Rahmati et al. [133], the amino group of the aptamer promoted covalent attachment to the 3D N-C@NiCo_2_O_4_ NWs nanocomposite and the aptasensor has been utilized for HCV detection. The very high surface to volume ratio, high density of metal sites and porosity of the nanocomposite potentially enable greater aptamer load onto the GCE surface. This was evident through increase in Rct value upon aptamer immobilization (Rct = 3.08 kΩ). The addition of increasing HCV concentration further increases the Rct value, indicating restricted electron transfer due to successful formation of HCV/aptamer complex on the aptasensor surface. Their aptasensor showed high sensitivity with detection limit of 0.16 fg/mL (Table 6) with excellent recovery in real samples of human blood serum [133].

**Figure 3:**
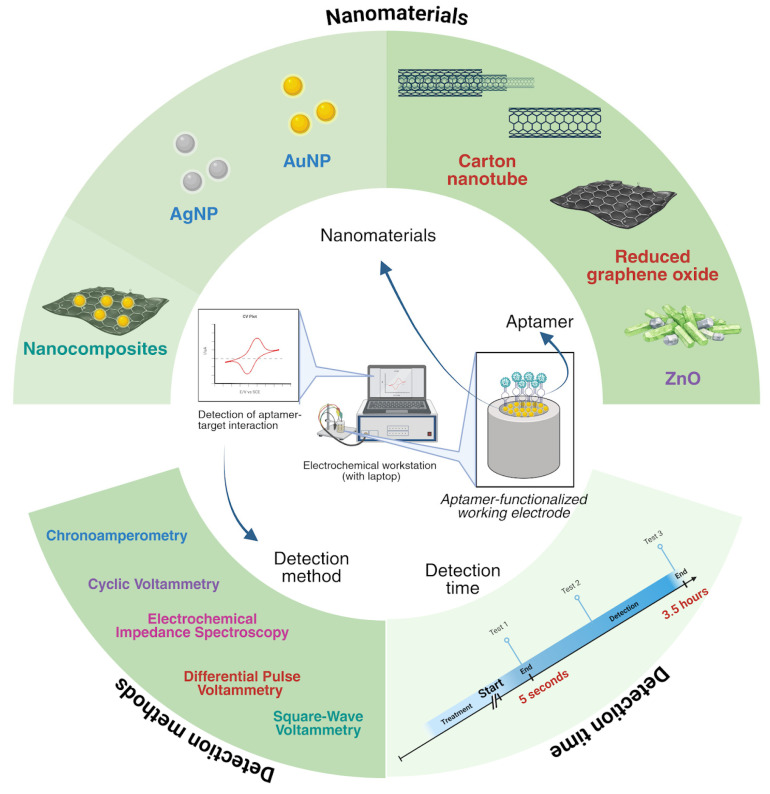
Overview of nanomaterials based electrochemical aptasensors that utilize different nanomaterials and methods for detection of pathogens and toxins and the time taken for the analysis.

It can be summarized that diverse types of nanomaterials have been utilized in electrochemical aptasensors research, aiming at providing sensitive, specific, and rapid detection of pathogenic bacteria, viruses, and by-products (toxins). The common detection methods include CV, DPV and EIS, though other methods have also been mentioned. The detection times vary depending on factors such as sample complexity, signal amplification, aptamer stability, etc. An overview of this review is presented in Figure 3.

## 4. Challenges and Future Perspectives

The use of nanomaterials on the electrode surface to enhance signal amplification of electrochemical aptasensors helps to overcome the limitations of inhibitory effects of the aptamer on electron transfer [135]. However, it is important to note that there are limitations and challenges in using nanomaterials for electrochemical aptasensors. Aptamer immobilization which is very crucial in aptasensor development may require chemical modification or functionalization of the nanomaterials or aptamer (e.g., thiol group) for interaction between aptamer and nanomaterials [102]. In addition, the orientation and biological activity of the immobilized aptamer must be maintained as inappropriate immobilization may lead to loss of activity, low biocompatibility, and less specificity [102], which hinder the optimum interaction of target–aptamer and thus may reduce the aptasensor sensitivity. Though large DNA loading is essential for sensitive detection, it must be kept at a minimal amount to facilitate correct folding and avoid steric hindrance [136]. Other challenges would be the production cost in which the metal nanomaterials may impose more cost compared to the carbon-based nanomaterials [84]. Sensitivity of the aptasensor could also be affected by the batch-to-batch variation in nanomaterials, for example, the sizes of metal nanoparticles which result in signal fluctuations [103,137]. These challenges limit widespread clinical adoption and commercialization of nanomaterial-based biosensors for diagnostic purposes.

An ideal on-site biosensor monitoring device should meet the required sensitivity, must be highly reproducible [102], and can withstand different conditions and complexity of samples. The presence of interfering compounds known as fouling agents in complex samples such as serum also limits the functionality and effectiveness of the electrochemical aptasensors. These agents can include a wide range of molecules such as proteins, nucleic acids, amino acids, neurotransmitters, and phenols, as well as whole cells and their fragments which tend to adhere to the electrode surface through adsorption, precipitation, or polymerization. The biofouling can lead to false readings or inhibit target binding on electrode surface, which in turn affect the sensitivity, stability, reproducibility, and overall reliability of the biosensors [138]. In addition, long-term stability of aptamers remains a challenge as their activity is highly dependent on the physiological conditions. Denaturation of aptamer could potentially occur during storage, shipping, or long-term monitoring of analytes, demanding better solutions for commercial use [139]. For practical applications, electrochemical sensors must attain sensitive and specific detection as well as capability for long-term and real-time monitoring [140].

Concerns involving biofouling remain despite the existence of several strategies that are compatible with electrochemical measurements such as utilization of nanoporous gold, zwitterionic polymers, hydrophilic polymeric membranes, etc. [140,141]. Therefore, to meet the market demand of having sensitive and robust devices for analytical measurements, future research could be directed towards designing effective antifouling sensing platforms that can reduce nonspecific adsorption and increase the signal-to-noise ratio. Recent studies have also investigated the potential integration of electrochemical aptasensors with microfluidics for detection of pathogens such as norovirus [47] and *Vibrio parahaemolyticus* [142], providing advantages of high sensitivity, low cost, rapid detection, and easy miniaturization. It is noteworthy that portable diagnostic devices with high sensitivity, specificity and reproducibility are strongly desired as an alternative to the conventional methods for detection of pathogens and the by-products intended for point-of-care (POC) diagnostics, food safety, and environmental monitoring. Looking forward, integration of artificial intelligence (AI) and microfluidic-integrated lab-on-a-chip (LOC) systems in the development of electrochemical aptasensors may offer advanced analysis with improved accuracy, encourage precision medicine and personalized clinical tools, permit automation, easy operation and real-time monitoring as well as reduce sample volume. Considering the limitations and challenges, advancement in technology could produce a rapid detection electrochemical aptasensor diagnostic kit that may potentially help reduce diagnostic time and lower the global disease burden.

## 5. Conclusions

Electrochemical aptasensors utilizing nanomaterials are promising cutting-edge solutions for rapid detection of infectious disease and environmental monitoring. Taken together, through the specific binding ability of aptamer which recognizes the whole pathogen or prominent surface structures, and the large specific surface areas, stability and good electrical conductivity of nanomaterials, the high sensitivity and specificity of electrochemical aptasensors can be achieved. This review revealed that the advancement in nanomaterials technology has enabled rapid detection of infectious agents and toxins using electrochemical aptasensors. Despite the limitations and challenges that exist, electrochemical aptasensors can be considered as an alternative to the conventional methods of culture-based methods, ELISA and PCR for early infection detection, diagnosis, and monitoring. The future developments may provide better outcomes through integration of electrochemical aptasensors with AI, microfluidics, and other systems.

## Figures and Tables

**Table 1:** Aptamers for detection of target bacteria and viruses.

Aptamer	Sequence of Aptamers	Target Bacteria/ Virus	Associated Disease	Reference
AP_7462DNA	5′-GCGGCGCGGTATGGAATTAGTGACTTCCGCGCGCCCCATTTTTTATAGGGGCCGC-3′	SARS-CoV-2 virus (spike protein)	COVID-19	[33]
SA43	5′-SHTCGGCACGTCAGTAGCGCTCGCTGGTCATCCCACAGCTACGTC-3′	*S. aureus*	Septicemia, sepsis, skin and soft tissue infections, endocarditis	[37]
Thiolated MTP64 aptamer	HS-(CH6)6-OP(O)2O-(CH2CH2O)6-5′-TTTTT-aptamer-3′	*M. tuberculosis* (MPT64 protein)	Tuberculosis	[38]
6011	5′-GCCTGTTGTGAGCCTCCTGTCGAACAACCACTCATATCTACTACATGACTTGCTCCATTCTGTTCTTTCTCTACGCATTGAGCGTTTATTCTTGTCTCCC-3′	Zaire Ebola virus (EBOV) soluble glycoprotein (sGP) and surface glycoprotein (GP1,2)	Hemorrhagic fever	[39]
LM6-116	5′-AGTATACGTATTACCTGCAGCTACTCGTTATTTCGTAGCACTTTTCCCCACCACCTTGGTGCGATATCTCGGAGATCTTGC-3′	*L. monocytogenes*	Sepsis, meningitis	[40]
apt 1; apt 2	5′-SH-AGTAATGCCCGGTAGTTATTCAAAGATGAGTAGGAAAAGA-3′ (apt 1); 5′-ROX-AGTAATGCCCGGTAGTTATTCAAA GATGAGTAGGAAAAGA-3′ (apt 2)	*Salmonella typhimurium* (whole cell)	Gastroenteritis	[41]
E-CA 20; E-CA 20P	5′-CACACACGGAACCCCGACAACATACATACGGTGAGGGTGG-3′; 5′-TTCACGGTAGCACGCATAGGCACACACGGAACCCCGACAACATACATACGGTGAGGGTGGCATCTGACCTCTGTGCTGCT-3′	*Streptococcus pyogenes* (M-type, M11 protein)	Pharyngitis, necrotizing fasciitis, sepsis, rheumatic fever, glomerulonephritis	[42]
P12-21, P12-55; P12-11, P12-31; P12-52a, P12-52b (clone)	CCGGAGGTGGGTGAGGTCTGCGGCAGGCTGTGTGGGTGGACCGGAGGGGGGTGAGGTCTGCGGCAGGCTGTGTGGGTGGA; CCCTCCGGGGGGGGGGGTCATCGGGATACCTGGTAAGGATACCCTCCGGGGGGG−−−−TCATCGGGATACCTGGTAAGGATA; CCGCCCAGCGGGGGTAGGGCCGGACGTAGGAGGAGCTGCG	*E. coli*, (whole cell)	Urinary tract infections, intestinal and diarrheal diseases, sepsis/meningitis	[43]
JN27	5′-ATGAGAGCGTCGGTGTGGTAACTAGTCTGATTTCTATTTCCTTTAATTAGTCTGCACACATTGCATTGTAGGAGGGTGCGGAAGTA-3′	*P. aeruginosa*	Cystic fibrosis, infections in burn wounds	[44]
HIV ssDNA aptamer	5′-NH2-GGGGGGCCAAGGCCCAGCCCTCACACA-3′	Human immunodeficiency virus (HIV)-1	Acquired immune deficiency syndrome (AIDS)	[45]
HCVcp ssDNA aptamer	-	Hepatitis C virus (HCV) (core protein)	Liver cirrhosis, hepatocarcinoma	[46]
Bt-Apt-Fc	5′-AGTATACCGTATTACCTGCAGCCATGTTTTGTAGGTGTAATAGGTCATGTTAGGGTTTCTGCGATATCTCGGAGATCTTGC-3′	Norovirus	Gastroenteritis	[47]
10	5′-GGCTGTTGTTGTTACCTATTGCGTGGCGATCGGACTTTCGATTCCGATTAACGCCGGAGG-3	Zika virus (NS1 protein)	Guillain–Barré syndrome (GBS), meningoencephalitis	[48]

**Table 2:** Metal nanoparticles-modified electrochemical aptasensors for detection of pathogens and by-products in various samples ^1^.

Target	Material	Method	LOD	Range	Detection Time	Detection Sample	Reference
*P. aeruginosa*	AuNPs	Amperometry	60 CFU/mL	60.0–6.0 × 10^7^ CFU/mL	10 min	Water	[89]
*Shigella* *dysenteriae*	AuNPs	EIS	10^0^ CFU/mL	10^1^–10^6^ CFU/mL	30 min	Water, skim milk	[90]
*Salmonella typhimurium*	AuNPs	DPV	16 CFU/mL	20–2 × 10^8^ CFU/mL	>3.5 h	Mineral water	[91]
H5N1 virus hemagglutinin (HA) protein	pAuNP	CV	1 pM	1 pM–100 nM	2 h	Chicken serum	[92]
SARS-CoV-2 virus spike (S) protein	AuNPs	DPV	91.1 pM	10 pM–6 nM	35 min	Artificial saliva, human serum	[93]
Zika virus	AuNPs	DPV	0.2 fM/ 33 fM	10–600 fM/ 500 fM–10 pM	N/A	Serum	[94]
Microcystin-LR (MC-LR), Cylindrospermopsin (CYL), anatoxin-α, saxitoxin and okadaic acid (OA)	AuNPs	SWV	0.0033 nM (MC-LR), 0.0045 nM (CYL), 0.0034 nM (anatoxin-α), 0.0053 nM (saxitoxin) and 0.0048 nM (OA)	0.073–150 nM (MC-LR) 0.018–200 nM (CYL, anatoxin-α, saxitoxin, OA)	20 min	Tap water	[95]
*P. aeruginosa*	AgNP	EIS	33 CFU/mL	10^2^–10^7^ CFU/mL	50 min	Human serum	[96]
*S. aureus*	AgNP	DPV	1 CFU/mL	10^1^–10^7^ CFU/mL	N/A	Water	[97]
Saxitoxin (STX)	AgNP	DPV	1 nM	0.04–0.15 µM	N/A	Clams, mantis shrimp	[98]
*Salmonella* *typhimurium*	NiNWs	EIS	80 CFU/mL	10^2^–10^6^ CFU/mL	2 h	Chicken meat	[99]

^1^ AgNPs: silver nanoparticles; AuNp; gold nanoparticles; CV = cyclic voltammetry; DPV = differential pulse voltammetry; EIS = electrochemical impedance spectroscopy; N/A: not available; NiNWs: nickel nanowires; pAuNP: porous gold nanoparticles; SWV: square wave voltammetry.

**Table 3:** Metal nanocomposite-modified electrochemical aptasensors for detection of pathogens and by-products in various samples ^1^.

Target	Material	Method	LOD	Range	Detection Time	Detection Sample	Reference
*S. aureus*	Ag-Cs-Gr QDs/ NTiO_2_	DPV	3.3 CFU/mL	10–5 × 10^8^ CFU/mL	90 min	Human serum	[11]
*S. aureus*	AuNPs/SPANI	EIS	2 CFU/mL	10–10^5^ CFU/mL	N/A	Water, milk	[103]
*S. aureus*	AuNPs/CNPs/ CNFs	EIS	1 CFU/mL	1.2 × 10– 1.2 × 10^8^ CFU/mL	N/A	Human serum	[104]
*M. tuberculosis* (antigen MPT64)	GNP-C_60_-PAn	DPV	20 fg/mL	0.02–1000 pg/mL	N/A	Human serums	[105]
*E. coli* O157:H7	AuNPs/rGO–PVA	EIS	9.34 CFU/mL	9.2–9.2 × 10^8^ CFU/mL	N/A	Tap water, milk, meat	[106]
*Helicobacter* *pylori* (Hsp 60)	Hap-Ag-ZnO	SWV	0.429 nM	0.05–300 nM	20 min	Human serum	[107]
*Listeria* *monocytogenes*	Pt/HCNs	DPV	2 CFU/mL	10–10^9^ CFU/mL	N/A	Milk, lettuce homogenate	[108]
Norovirus (NoV)	BP-AuNCs	DPV	0.28 ng/mL	1 ng/mL–10 µg/mL	30 min	Oyster	[109]
T-2 toxin (mycotoxin)	MoS_2_-PANI-Chi-Au and rGO-TEPA-Au@Pt NRs	Chronoa-mperome-try	1.79 fg/mL	10 fg/mL–100 ng/mL	N/A	Beer	[110]
Staphylococcal Enterotoxin A (SEA)	AuNUs/rGO	DPV	7.6 fM	25.0–950.0 fM	100 min	Milk, meat extract, human serum	[111]

^1^ Ag-Cs-Gr QDs/NTiO_2_: silver nanoparticles–chitosan–graphene quantum dots/nitrogen-doped TiO_2_ nanoparticles; AuNUs/rGO: gold nano urchins/reduced graphene oxide; AuNPs/CNPs/CNFs: gold nanoparticles/carbon nanoparticles/cellulose nanofibers nanocomposite; AuNPs/SPANI: gold nanoparticles/sulfonated polyaniline; BP-AuNCs: phosphorene–gold nanocomposites; GNP-C60-PAn: gold nanoparticles decorated with coil-like fullerene-doped polyaniline; Hap-Ag-ZnO: hydroxyapatite–silver–zinc oxide; MoS2-PANI-Chi-Au: molybdenum disulfide–polyaniline–chitosan–gold nanoparticles; N/A: not available; Pt/HCNs: Platinum nanoparticles/hollow carbon nanospheres; rGO-TEPA-Au@Pt NRs: Reduced graphene oxide–tetraethylene pentamine–gold@platinum nanorods.

**Table 4:** Metal/metal oxide nanocomposite-modified electrochemical aptasensors for detection of pathogens and by-products in various samples ^1^.

Target	Material	Method	LOD	Range	Detection Time	Detection Sample	Reference
Chikungunya virus antigen (CHIKV-Ag)	Ag-Au-ZnO	CV	1 ng/mL	1 ng/mL–10 µg/mL	20 s	Human serum	[115]
*S. aureus*	AuNPs@Fe_3_O_4_	DPV	1 CFU/mL	10^1^–10^7^ CFU mL^−1^	N/A	Milk, conduit water, apple juice	[116]
T-2 toxin	Au/(Ce-In)Ox	DPV	7.6 × 10^−8^ ng/mL	5.0 × 10^−7^ ng/mL–5.0 ng/mL	N/A	Maize	[117]
Ochratoxin A	ZnO-Au	DPV	0.05 pg/mL	0.1–30,000 pg/mL	N/A	Wine and beer	[118]

^1^ Ag-Au-ZnO: silver nanoparticles–gold nanoparticles–zinc oxide nanorods; Au/(Ce-In)Ox: gold nanoparticles/bimetallic oxide (cerium–indium); AuNPs@Fe_3_O_4_: gold nanoparticles modified magnetic nanoparticles; CV: cyclic voltammetry; DPV: differential pulse voltammetry; N/A: not available; ZnO-Au: Zinc oxide–gold nanoparticles.

**Table 5:** Carbon nanomaterials-modified electrochemical aptasensors for detection of bacteria in various samples ^1^.

Target	Material	Method	LOD	Range	Detection Time	Detection Sample	Reference
*E. coli* O78:K80:H11	BRG	EIS	10^1^ CFU/mL	10^1^–10^6^ CFU/mL	≤8 min	Water, juice, and milk	[121]
*Salmonella enteritidis*/ *Salmonella typhimurium*	MWCNTs	EIS	5.5 × 10^1^ CFU/mL/ 6.7 × 10^1^ CFU/mL	5.5 × 10^1^–5.5 × 10^6^ CFU/mL/ 6.7 × 10^1^–6.7 × 10^5^ CFU/mL	20 min	Raw chicken meat	[122]

^1^ BRG: bridged rebar graphene, EIS: electrochemical impedance spectroscopy; MWCNTs: multi-walled carbon nanotubes.

**Table 6:** Carbon nanocomposites-modified electrochemical aptasensors for detection of pathogens and by-products in various samples ^1^.

Target	Material	Method	LOD	Range	Detection Time	Detection Sample	Reference
*Acinetobacter baumannii*	rGO/MWCNT/CS/CQD	DPV	1 CFU/mL	10–1 × 10^7^ CFU/mL	N/A	Serum, Skimmed milk	[25]
*S. aureus*	NCNO/AuNPs	EIS	3 CFU/mL	10^1^–10^8^ CFU/mL	15 min	Human serum	[37]
*M. tuberculosis*	RGO/PNE/Au	LSV	0.1 × 10^−7^ μM	0.1 × 10^−2^–0.1 × 10^−7^ μM	5 s	N/A	[83]
*Salmonella* *Typhi* (Vi antigen)	MoS_2_-rGO	SWV	100 pg/mL	0.1–1000 ng/mL	N/A	Sera and urine	[123]
*M. tuberculosis* (antigen ESAT-6)	P-MOF-rGO/Pt@Au	CV	3.3 × 10^−5^ ng/mL	1.0 × 10^−4^– 2.0 × 10^2^ ng/mL	1 h	Human serum	[124]
*Salmonella* *Typhimurium*	rGO-AP	DPV	10^1^ CFU/mL	10^1^–10^8^ CFU/mL	N/A	Chicken meat	[125]
*Salmonella* *Typhimurium*	rGO-CHI	DPV	10^1^ CFU/mL	10^1^–10^6^ CFU/mL	N/A	Chicken meat	[126]
*Salmonella* *Typhimurium*	rGO-TiO_2_	DPV	10^1^ CFU/mL	10^1^–10^8^ CFU/mL	1 h	Chicken meat	[127]
*Salmonella* *Typhimurium*	rGO-CNT	DPV	10^1^ CFU/mL	10^1^–10^8^ CFU/mL	10 min	Chicken meat	[128]
*E. coli* (LPS)	rGO/AuNPs	EIS	30 fg/mL/1 fg/mL	N/A	35 min	Human serum	[129]
Lipopolysaccharide (LPS)/endotoxin	MRGO-Au	SWV	4 fg/mL/ 0.2 fg/mL	0.1–0.9 pg/mL/ 0.01–0.09 pg/mL	>35 min	Human blood serum	[130]
Hepatitis B virus surface antigen (HBsAg)	rGO-AuNPs	CV	0.0014 fg/mL	0.125–2.0 fg/mL	N/A	Human serum	[131]
Hepatitis C virus core antigen (Anti)	MWCNTs- Chit	DPV	1.67 fg/mL	5.0 fg/mL– 1.0 pg/mL	N/A	Human serum	[132]
Hepatitis C virus core antigen	3D N-C@NiCo_2_O_4_ NWs	EIS	0.16 fg/mL	0.5 fg/mL –0.12 pg/mL	N/A	Human blood serum	[133]
human papillomavirus (HPV-16 L1 protein)	prGO-MoS_2_	DPV	0.1 ng/mL	0.2–2 ng/mL	N/A	Human serum and saliva	[134]

^1^ CV: cyclic voltammetry; CS/CQD: chitosan/carbon quantum dot; DPV: differential pulse voltammetry; EIS: electrochemical impedance spectroscopy; LPS: lipopolysaccharide; LSV: linear sweep voltammetry; MoS_2_-rGO: Molybdenum disulfide-reduced graphene oxide; MRGO-Au: magnetite-reduced graphene oxide with gold nanoparticles; MWCNTs-Chit: multi-walled carbon nanotubes–chitosan nanocomposite; N/A: not available; NCNO/AuNPs: nitrogen-doped carbon nano-onions/gold nanoparticles; rGO-AP: rGO-azophloxine; rGO-AuNPs: reduced graphene oxide–gold nanoparticles; rGO-CHI: reduced graphene oxide–chitosan; rGO-CNT: reduced graphene oxide–carbon nanotube; RGO/PNE/Au: reduced graphene oxide/polynorepinephrine/gold nanoparticles; rGO-TiO_2_: reduced graphene oxide–titanium dioxide; prGO-MoS_2_: porous reduced graphene oxide–molybdenum sulfide; Pt@Au: platinum aurum core shell nanoparticles; P-MOF-rGO: poly (diallyldimethylammonium chloride)–metal–organic framework doped reduced graphene oxide; SWV: square wave voltammetry; 3D N-C@NiCo_2_O_4_ NWs: N-doped carbon@ NiCo_2_O_4_ nanowires.

## Data Availability

No new data were created or analyzed in this study. Data sharing is not applicable to this article.

## References

[1] Zhang Z., Zhou J., Du X. (2019). Electrochemical biosensors for detection of foodborne pathogens. Micromachines.

[2] Li H.Y., Jia W.N., Li X.Y., Zhang L., Liu C., Wu J. (2020). Advances in detection of infectious agents by aptamer-based technologies. Emerg. Microbes Infect..

[3] Kaur H., Shorie M. (2019). Nanomaterial based aptasensors for clinical and environmental diagnostic applications. Nanoscale Adv..

[4] Wu W., Yu C., Wang Q., Zhao F., He H., Liu C., Yang Q. (2020). Research advances of DNA aptasensors for foodborne pathogen detection. Crit. Rev. Food Sci. Nutr..

[5] Al-Rohaimi A.H., Al Otaibi F. (2020). Novel SARS-CoV-2 outbreak and COVID19 disease; a systemic review on the global pandemic. Genes Dis..

[6] Sah R., Mohanty A., Reda A., Siddiq A., Mohapatra R.K., Dhama K. (2022). Marburg virus re-emerged in 2022: Recently detected in Ghana, another zoonotic pathogen coming up amid rising cases of Monkeypox and ongoing COVID-19 pandemic- global health concerns and counteracting measures. Vet. Q..

[7] Goluch E.D. (2017). Microbial identification using electrochemical detection of metabolites. Trends Biotechnol..

[8] Nouri A., Ahari H., Shahbazzadeh D. (2018). Designing a direct ELISA kit for the detection of *Staphylococcus aureus* enterotoxin A in raw milk samples. Int. J. Biol. Macromol..

[9] Rajapaksha P., Elbourne A., Gangadoo S., Brown R., Cozzolino D., Chapman J. (2019). A review of methods for the detection of pathogenic microorganisms. Analyst.

[10] Shan Y., Xu C., Wang M., Zhu Z., Wu F.-G., Shi Z., Cui Q., Arumugam G.M. (2019). Bilinear *Staphylococcus aureus* detection based on suspension immunoassay. Talanta.

[11] Ghalkhani M., Sohouli E., Khaloo S.S., Vaziri M.H. (2022). Architecting of an aptasensor for the *Staphylococcus aureus* analysis by modification of the screen-printed carbon electrode with aptamer/Ag–Cs-Gr QDs/NTiO_2_. Chemosphere.

[12] Zhou B., Ye Q., Chen M., Li F., Xiang X., Shang Y., Wang C., Zhang J., Xue L., Wang J. (2022). Novel species-specific targets for real-time PCR detection of four common pathogenic *Staphylococcus spp*. Food Control.

[13] Hudu S.A., Alshrari A.S., Syahida A., Sekawi Z. (2016). Cell culture, technology: Enhancing the culture of diagnosing human diseases. J. Clin. Diagn. Res..

[14] Simoska O., Stevenson K.J. (2019). Electrochemical sensors for rapid diagnosis of pathogens in real time. Analyst.

[15] Satija J., Punjabi N., Mishra D., Mukherji S. (2016). Plasmonic-ELISA: Expanding horizons. RSC Adv..

[16] Hosseini S., Vázquez-Villegas P., Rito-Palomares M., Martinez-Chapa S.O. (2018). Advantages, disadvantages and modifications of conventional ELISA. Enzyme-Linked Immunosorbent Assay (ELISA).

[17] Wu L., Li G., Xu X., Zhu L., Huang R., Chen X. (2019). Application of nano-ELISA in food analysis: Recent advances and challenges. TrAC Trends Anal. Chem..

[18] Lee W.L., Gu X., Armas F., Chandra F., Chen H., Wu F., Leifels M., Xiao A., Chua F.J.D., Kwok G.W.C. (2021). Quantitative SARS-CoV-2 tracking of variants Delta, Delta plus, Kappa and Beta in wastewater by allele-specific RT-qPCR. Environ. Sci. Technol. Lett..

[19] Vidic J., Vizzini P., Manzano M., Kavanaugh D., Ramarao N., Zivkovic M., Radonic V., Knezevic N., Giouroudi I., Gadjanski I. (2019). Point-of-need DNA testing for detection of foodborne pathogenic bacteria. Sensors.

[20] Zhao W., Zhang D., Zhou T., Huang J., Wang Y., Li B., Chen L., Yang J., Liu Y. (2022). Aptamer-conjugated magnetic Fe_3_O_4_@Au core-shell multifunctional nanoprobe: A three-in-one aptasensor for selective capture, sensitive SERS detection and efficient near-infrared light triggered photothermal therapy of *Staphylococcus aureus*. Sens. Actuators B Chem..

[21] Castillo-Henríquez L., Brenes-Acuña M., Castro-Rojas A., Cordero-Salmerón R., Lopretti-Correa M., Vega-Baudrit J.R. (2020). Biosensors for the detection of bacterial and viral clinical pathogens. Sensors.

[22] Haleem A., Javaid M., Singh R.P., Suman R., Rab S. (2021). Biosensors applications in medical field: A brief review. Sens. Int..

[23] Chadha U., Bhardwaj P., Agarwal R., Rawat P., Agarwal R., Gupta I., Panjwani M., Singh S., Ahuja C., Selvaraj S.K. (2022). Recent progress and growth in biosensors technology: A critical review. J. Ind. Eng. Chem..

[24] Feng X., Li P., Li T., Cao X., Liu D., Xiao M., Wang L. (2024). Ultra-sensitive and rapid detection of *Salmonella enterica* and *Staphylococcus aureus* to single-cell level by aptamer-functionalized carbon nanotube field-effect transistor biosensors. Biosens. Bioelectron..

[25] Abedi R., Raoof J.B., Mohseni M., Hashkavayi A.B. (2024). Sandwich-type electrochemical aptasensor based on hemin-graphite oxide as a signal label and rGO/MWCNTs/chitosan/carbon quantum dot modified electrode for sensitive detection of *Acinetobacter baumannii* bacteria. Anal. Chim. Acta.

[26] Tepeli Y., Anik Ü. (2018). Electrochemical biosensors for influenza virus A detection: The potential of adaptation of these devices to POC systems. Sens. Actuators B Chem..

[27] Li D., Liu L., Huang Q., Tong T., Zhou Y., Li Z., Bai Q., Liang H., Chen L. (2021). Recent advances on aptamer-based biosensors for detection of pathogenic bacteria. World J. Microbiol. Biotechnol..

[28] Napit R., Jaysawal S.K., Chowdhury R., Catague J., Melke H., Pham C.V., Xu H., Jia L., Lin J., Hou Y. (2025). Aptasensors and advancement in molecular recognition technology. Adv. Mater. Technol..

[29] Sargazi S., Simge E.R., Mobashar A., Gelen S.S., Rahdar A., Ebrahimi N., Hosseinikhah S.M., Bilal M., Kyzas G.Z. (2022). Aptamer-conjugated carbon-based nanomaterials for cancer and bacteria theranostics: A review. Chem. Biol. Interact..

[30] Léguillier V., Heddi B., Vidic J. (2024). Recent advances in aptamer-based biosensors for bacterial detection. Biosensors.

[31] Lee S.J., Cho J., Lee B.H., Hwang D., Park J.W. (2023). Design and prediction of aptamers assisted by in silico methods. Biomedicines.

[32] Ishida R., Adachi T., Yokota A., Yoshihara H., Aoki K., Nakamura Y., Hamada M. (2020). RaptRanker: In silico RNA aptamer selection from HT-SELEX experiment based on local sequence and structure information. Nucleic Acids Res..

[33] Cossettini A., Pasquardini L., Romani A., Feriani A., Pinamonti D., Manzano M. (2024). Computational aptamer design for spike glycoprotein (S) (SARS CoV-2) detection with an electrochemical aptasensors. Appl. Microbiol. Biotechnol..

[34] Song Y., Song J., Wei X., Huang M., Sun M., Zhu L., Lin B., Shen H., Zhu Z., Yang C. (2020). Discovery of aptamers targeting the receptor-binding domain of the SARS-CoV-2 spike glycoprotein. Anal. Chem..

[35] Abrego-Martinez J.C., Jafari M., Chergui S., Pavel C., Che D., Siaj M. (2022). Aptamer-based electrochemical biosensor for rapid detection of SARS-CoV-2: Nanoscale electrode-aptamer-SARS-CoV-2 imaging by photo-induced force microscopy. Biosens. Bioelectron..

[36] Wandtke T., Wędrowska E., Szczur M., Przybylski G., Libura M., Kopiński P. (2022). Aptamers—Diagnostic and therapeutic solution in SARS-CoV-2. Int. J. Mol. Sci..

[37] Sohouli E., Ghalkhani M., Zargar T., Joseph Y., Rahimi-Nasrabadi M., Ahmadi F., Plonska-Brzezinska M.E., Ehrlich H. (2022). A new electrochemical aptasensor based on gold/nitrogen-doped carbon nano-onions for the detection of *Staphylococcus aureus*. Electrochim. Acta.

[38] Sypabekova M., Jolly P., Estrela P., Kanayeva D. (2019). Electrochemical aptasensor using optimized surface chemistry for the detection of *Mycobacterium tuberculosis* secreted protein MPT64 in human serum. Biosens. Bioelectron..

[39] Banerjee S., Hemmat M.A., Shubham S., Gosai A., Devarakonda S., Jiang N., Geekiyanage C., Dillard J.A., Maury W., Shrotriya P. (2023). Structurally different yet functionally similar: Aptamers specific for the Ebola virus soluble glycoprotein and GP1,2 and their application in electrochemical sensing. Int. J. Mol. Sci..

[40] Suh S.H., Choi S.J., Dwivedi H.P., Moore M.D., Escudero-Abarca B.I., Jaykus L.A. (2018). Use of DNA aptamer for sandwich type detection of *Listeria monocytogenes*. Anal. Biochem..

[41] Duan N., Chang B., Zhang H., Wang Z., Wu S. (2016). *Salmonella typhimurium* detection using a surface-enhanced Raman scattering-based aptasensors. Int. J. Food Microbiol..

[42] Hamula C.L.A., Peng H., Wang Z., Tyrrell G.J., Li X.-F., Le X.C. (2016). An improved SELEX technique for selection of DNA aptamers binding to M-type 11 of *Streptococcus pyogenes*. Methods.

[43] Marton S., Cleto F., Krieger M.A., Cardoso J. (2016). Isolation of an aptamer that binds specifically to *E. coli*. PLoS ONE.

[44] Soundy J., Day D. (2017). Selection of DNA aptamers specific for live *Pseudomonas aeruginosa*. PLoS ONE.

[45] Babamiri B., Salimi A., Hallaj R. (2018). A molecularly imprinted electrochemiluminescence sensor for ultrasensitive HIV-1 gene detection using EuS nanocrystals as luminophore. Biosens. Bioelectron..

[46] Li X., Yin C., Wu Y., Zhang Z., Jiang D., Xiao D., Fang X., Zhou C. (2020). Plasmonic nanoplatform for point-of-care testing trace HCV core protein. Biosens. Bioelectron..

[47] Chand R., Neethirajan S. (2017). Microfluidic platform integrated with graphene-gold nano-composite aptasensor for one-step detection of norovirus. Biosens. Bioelectron..

[48] Lee K.H., Zeng H. (2017). Aptamer-based ELISA assay for highly specific and sensitive detection of Zika NS1 protein. Anal. Chem..

[49] Chidzwondo F., Mutapi F. (2024). Challenge of diagnosing acute infections in poor resource settings in Africa. AAS Open Res. Afr..

[50] Fàbrega A., Vila J. (2013). *Salmonella enterica* serovar *Typhimurium* skills to succeed in the host: Virulence and regulation. Clin. Microbiol..

[51] Vallés P.G., Gil L.A.F., Cacciamani V., Benardon M.E., Costantino V.V. (2023). Shiga toxin-producing *Escherichia coli* associated hemolytic uremic syndrome. J. Urol. Nephrol..

[52] Jin T., Mohammad M., Pullerits R., Ali A. (2021). Bacteria and host interplay in *Staphylococcus aureus* septic arthritis and sepsis. Pathogens.

[53] Hmar E.B.L., Paul S., Sharma H.K. (2024). The role of *Shigella spp.* in propagating bacillary dysentery in humans and the prominence of nanotechnology in disease prevention. Future J. Pharm. Sci..

[54] Wasik D., Mulchandani A., Yates M.V. (2017). A heparin-functionalized carbon nanotube-based affinity biosensor for dengue virus. Biosens. Bioelectron..

[55] Mittler E., Dieterle M.E., Kleinfelter L.M., Slough M.M., Chandran K., Jangra R.K. (2019). Hantavirus entry: Perspectives and recent advances. Adv. Virus Res..

[56] Kharsany A.B.M., McKinnon L.R., Lewis L., Cawood C., Khanyile D., Maseko D.V., Goodman T.C., Beckett S., Govender K., George G. (2020). Population prevalence of sexually transmitted infections in a high HIV burden district in KwaZulu-Natal, South Africa: Implications for HIV epidemic control. Int. J. Infect. Dis..

[57] Rojas M., Monsalve D.M., Pacheco Y., Acosta-Ampudia Y., Ramírez-Santana C., Ansari A.A., Gershwin M.E., Anaya J.M. (2020). Ebola virus disease: An emerging and re-emerging viral threat. J. Autoimmun..

[58] Yin T.-L., Chen N., Zhang J.-Y., Yang S., Li W.-M., Gao X.-H., Shi H.-L., Hu H.-P. (2024). Excess multi-cause mortality linked to Influenza virus infection in China, 2012–2021: A population-based study. Front. Public Health.

[59] Msemburi W., Karlinsky A., Knutson V., Aleshin-Guendel S., Chatterji S., Wakefield J. (2023). The WHO estimates of excess mortality associated with the COVID-19 pandemic. Nature.

[60] Faramarzi A., Norouzi S., Dehdarirad H., Aghlmand S., Yusefzadeh H., Javan-Noughabi J. (2024). The global economic burden of COVID-19 disease: A comprehensive systematic review and meta-analysis. Syst. Rev..

[61] Scott A., Ansari W., Khan F., Chambers R., Benigno M., Di Fusco M., McGrath L., Malhotra D., Draica F., Nguyen J. (2024). Substantial health and economic burden of COVID-19 during the year after acute illness among US adults at high risk of severe COVID-19. BMC Med..

[62] Bengoechea J.A., Bamford C.G. (2020). SARS-CoV-2, bacterial co-infections, and AMR: The deadly trio in COVID-19. EMBO Mol. Med..

[63] Rawson T.M., Moore L.S.P., Zhu N., Ranganathan N., Skolimowska K., Gilchrist M., Satta G., Cooke G., Holmes A. (2020). Bacterial and fungal coinfection in individuals with coronavirus: A rapid review to support COVID-19 antimicrobial prescribing. Clin. Infect. Dis..

[64] Hendaus M.A., Jomha F.A. (2020). Covid-19 induced superimposed bacterial infection. J. Biomol. Struct. Dyn..

[65] Almand E.A., Moore M.D., Jaykus L.A. (2017). Virus-bacteria interactions: An emerging topic in human infection. Viruses.

[66] Mirzaei R., Goodarzi P., Asadi M., Soltani A., Aljanabi H.A.A., Jeda A.S., Dashtbin S., Jalalifar S., Mohammadzadeh R., Teimoori A. (2020). Bacterial co-infections with SARS-CoV-2. IUBMB Life.

[67] Thornhill J.P., Barkati S., Walmsley S., Rockstroh J., Antinori A., Harrison L.B., Palich R., Nori A., Reeves I., Habibi M.S. (2022). Monkeypox virus infection in humans across 16 Countries—April-June 2022. N. Engl. J. Med..

[68] Marburg Virus Disease. https://www.who.int/news-room/fact-sheets/detail/marburg-virus-disease.

[69] Marburg Virus Disease—Ghana. https://www.who.int/emergencies/disease-outbreak-news/item/2022-don402.

[70] Zhao F., He Y., Lu H. (2022). Marburg virus disease: A deadly rare virus is coming. Biosci. Trends.

[71] Ebola Virus Disease. https://www.who.int/news-room/fact-sheets/detail/ebola-disease.

[72] Keita A.K., Koundouno F.R., Faye M., Düx A., Hinzmann J., Diallo H., Ayouba A., Marcis F.L., Soropogui B., Ifono K. (2021). Resurgence of Ebola virus in 2021 in Guinea suggests a new paradigm for outbreaks. Nature.

[73] Xiong W.M., Xu Q.P., Li X., Xiao R.D., Cai L., He F. (2017). The association between human papillomavirus infection and lung cancer: A system review and meta-analysis. Oncotarget.

[74] Yang X., Qi S., Dai L., Ye Q., Li X. (2025). Trends in HPV-positive cervical cancer prevalence: A retrospective study from 2013 to 2020. Virol. J..

[75] Zhang C.-H., Cheng Y., Zhang S., Fan J., Gao Q. (2022). Changing epidemiology of hepatocellular carcinoma in Asia. Liver Int..

[76] Sung H., Ferlay J., Siegel R.L., Laversanne M., Soerjomataram I., Jemal A., Bray F. (2021). Global Cancer Statistics 2020: GLOBOCAN estimates of incidence and mortality worldwide for 36 cancers in 185 countries. CA Cancer J. Clin..

[77] Martins I., Ribeiro I.P., Jorge J., Gonçalves A.C., Gonçalves A.C., Melo J.B., Carreira I.M. (2021). Liquid biopsies: Applications for cancer diagnosis and monitoring. Genes.

[78] Li F., Yu Z., Han X., Lai R.Y. (2019). Electrochemical aptamer-based sensors for food and water analysis: A review. Anal. Chim. Acta.

[79] Chen X., Gao D., Sun F., Li Z., Wang Y., Qiu C., He K., Wang J. (2022). Nanomaterial-based aptamer biosensors for ochratoxin A detection: A review. Anal. Bioanal. Chem..

[80] Choi H.K., Lee J., Park M., Oh J.H. (2017). Development of single-walled carbon nanotube-based biosensor for the detection of *Staphylococcus aureus*. J. Food Qual..

[81] Fatemi K., Lau S.Y., Obayomi K.S., Kiew S.F., Coorey R., Chung L.Y., Fatemi R., Heshmatipour Z., Premarathna K.S.D. (2024). Carbon nanomaterial-based aptasensors for rapid detection of foodborne pathogenic bacteria. Anal. Biochem..

[82] Jalalvand A.R., Karami M.M. (2025). Roles of nanotechnology in electrochemical sensors for medical diagnostic purposes: A review. Sens. Bio Sens. Res..

[83] Bisht N., Patel M., Dwivedi N., Kumar P., Mondal D.P., Srivastava A.K., Dhand C. (2023). Bio-inspired polynorepinephrine based nanocoatings for reduced graphene oxide/gold nanoparticles composite for high-performance biosensing of *Mycobacterium tuberculosis*. Environ. Res..

[84] Gómez-López P., Puente-Santiago A., Castro-Beltrán A., do Nascimento L.A.S., Balu A.M., Luque R., Alvarado-Beltrán C.G. (2020). Nanomaterials and catalysis for green chemistry. Curr. Opin. Green Sustain. Chem..

[85] Hammami I., Alabdallah N.M., Al Jomaa A., Kamoun M. (2021). Gold nanoparticles: Synthesis properties and applications. J. King Saud Univ. Sci..

[86] Kalimuthu K., Cha B.S., Kim S., Park K.S. (2020). Eco-friendly synthesis and biomedical applications of gold nanoparticles: A review. Microchem. J..

[87] Fahim M., Shahzaib A., Nishat N., Jahan A., Bhat T.A., Inam A. (2024). Green synthesis of silver nanoparticles: A comprehensive review of methods, influencing factors, and applications. JCIS Open.

[88] Philip D. (2010). Green synthesis of gold and silver nanoparticles using *Hibiscus rosa sinensis*. Phys. E Low Dimens. Syst. Nanostruct..

[89] Das R., Dhiman A., Kapil A., Bansal V., Sharma T.K. (2019). Aptamer-mediated colorimetric and electrochemical detection of Pseudomonas aeruginosa utilizing peroxidase-mimic activity of gold NanoZyme. Anal. Bioanal. Chem..

[90] Zarei S.S., Soleimanian-Zad S., Ensafi A.A. (2018). An impedimetric aptasensor for *Shigella dysenteriae* using a gold nanoparticle-modified glassy carbon electrode. Microchim. Acta.

[91] Ge C., Yuan R., Yi L., Yang J., Zhang H., Li L., Nian W., Yi G. (2018). Target-induced aptamer displacement on gold nanoparticles and rolling circle amplification for ultrasensitive live *Salmonella typhimurium* electrochemical biosensing. J. Electroanal. Chem..

[92] Lee T., Park S.Y., Jang H., Kim G.-H., Lee Y., Park C., Mohammadniaei M., Lee M.-H., Min J. (2019). Fabrication of electrochemical biosensor consisted of multi-functional DNA structure/porous Au nanoparticle for avian influenza virus (H5N1) in chicken serum. Mater. Sci. Eng. C.

[93] Khan R., Deshpande A.S., Proteasa G., Andreescu S. (2024). Aptamer-based electrochemical biosensor with S protein binding affinity for COVID-19 detection: Integrating computational design with experimental validation of S protein binding affinity. Sens. Actuators B Chem..

[94] Cajigas S., Alzate D., Orozco J. (2020). Gold nanoparticle/DNA-based nanobioconjugate for electrochemical detection of Zika virus. Microchim. Acta.

[95] Rhouati A., Zourob M. (2024). Development of a multiplexed electrochemical aptasensor for the detection of cyanotoxins. Biosensors.

[96] Roushani M., Sarabaegi M., Pourahmad F. (2019). Impedimetric aptasensor for *Pseudomonas aeruginosa* by using a glassy carbon electrode modified with silver nanoparticles. Microchim. Acta.

[97] Abbaspour A., Norouz-Sarvestani F., Noori A., Soltani N. (2015). Aptamer-conjugated silver nanoparticles for electrochemical dual-aptamer-based sandwich detection of *Staphylococcus aureus*. Biosens. Bioelectron..

[98] Zeng W., Tang X., Wu T., Han B., Wu L. (2024). Development of a highly sensitive aptamer-based electrochemical sensor for detecting saxitoxin based on K_3_Fe(CN)_6_ regulated silver nanoparticles. Anal. Chim. Acta.

[99] Wang L., Huo X., Qi W., Xia Z., Li Y., Lin J. (2020). Rapid and sensitive detection of *Salmonella typhimurium* using nickel nanowire bridge for electrochemical impedance amplification. Talanta.

[100] Jamal R.B., Gosewinkel U.B., Ferapontova E.E. (2024). Electrocatalytic aptasensor for bacterial detection exploiting ferricyanide reduction by methylene blue on mixed PEG/aptamer monolayers. Bioelectrochemistry.

[101] Li N., Huang X., Sun D., Yu W., Tan W., Luo Z., Chen Z. (2018). Dual-aptamer-based voltammetric biosensor for the *Mycobacterium tuberculosis* antigen MPT64 by using a gold electrode modified with a peroxidase loaded composite consisting of gold nanoparticles and a Zr(IV)/terephthalate metal-organic framework. Microchim. Acta.

[102] Cho I.H., Kim D.H., Park S. (2020). Electrochemical biosensors: Perspective on functional nanomaterials for on-site analysis. Biomat. Res..

[103] Gao H., Xu T., Zhou J., Rojas O., He M., Ji X., Dai H. (2022). Electrochemical sensing of *Staphylococcus aureus* based on conductive anti-fouling interface. Microchim. Acta.

[104] Ranjbar S., Shahrokhian S. (2018). Design and fabrication of an electrochemical aptasensor using Au nanoparticles/carbon nanoparticles/cellulose nanofibers nanocomposite for rapid and sensitive detection of *Staphylococcus aureus*. Bioelectrochemistry.

[105] Bai L., Chen Y., Bai Y., Chen Y., Zhou J., Huang A. (2017). Fullerene-doped polyaniline as new redox nanoprobe and catalyst in electrochemical aptasensor for ultrasensitive detection of *Mycobacterium tuberculosis* MPT64 antigen in human serum. Biomaterials.

[106] Qaanei M., Taheri R.A., Eskandari K. (2021). Electrochemical aptasensor for *Escherichia coli* O157:H7 bacteria detection using a nanocomposite of reduced graphene oxide, gold nanoparticles and polyvinyl alcohol. Anal. Methods.

[107] Rafique S., Akram R., Nasir R., Naz N., Shafique A., Bashir S., Haq Z. (2024). An aptamer and hydroxyapatite-silver-zinc oxide–based novel electrochemical sensor for ultrasensitive *H. pylori detection*. Microchim. Acta.

[108] Jiang X., Lv Z., Rao C., Chen X., Zhang Y., Lin F. (2023). Simple and highly sensitive electrochemical detection of *Listeria monocytogenes* based on aptamer-regulated Pt nanoparticles/hollow carbon spheres nanozyme activity. Sens. Actuators B Chem..

[109] Jiang H., Sun Z., Zhang C., Weng X. (2022). 3D-architectured aptasensor for ultrasensitive electrochemical detection of norovirus based on phosphorene-gold nanocomposites. Sens. Actuators B Chem..

[110] Zhong H., Yu C., Gao R., Chen J., Yu Y., Geng Y., Wen Y., He J. (2019). A novel sandwich aptasensor for detecting T-2 toxin based on rGO-TEPA-Au@Pt nanorods with a dual signal amplification strategy. Biosens. Bioelectron..

[111] Nodoushan S.M., Nasirizadeh N., Sedighian H., Kachuei R., Azimzadeh-Taft M., Fooladi A.A.I. (2022). Detection of Staphylococcal Enterotoxin A (SEA) using a sensitive nanomaterial-based electrochemical aptasensor. Diam. Relat. Mater..

[112] Nikolova M.P., Chavali M.S. (2020). Metal oxide nanoparticles as biomedical materials. Biomimetics.

[113] Zhang H., Yao S., Song X., Xu K., Wang J., Li J., Zhao C., Jin M. (2021). One-step colorimetric detection of *Staphylococcus aureus* based on target-induced shielding against the peroxidase mimicking activity of aptamer-functionalized gold-coated iron oxide nanocomposites. Talanta.

[114] Jiang D., Ni D., Rosenkrans Z.T., Huang P., Yan X., Cai W. (2019). Nanozyme: New horizons for responsive biomedical applications. Chem. Soc. Rev..

[115] Sharma P., Hassan H., Hasan M.R., Fatima T., Mosina K.M., Shukla S.K., Narang J. (2024). Enhanced electrochemical detection of chikungunya virus through aptasensor modification with origami paper-based ternary nanocomposite. Microchem. J..

[116] El-Wekil M.M., Halby H.M., Darweesh M., Ali M.E., Ali R. (2022). An innovative dual recognition aptasensor for specific detection of *Staphylococcus aureus* based on Au/Fe_3_O_4_ binary hybrid. Sci. Rep..

[117] Ming P., Lai H., Liu Y., Wang J., You F., Sun D., Zhai H. (2023). Aptasensor development for T-2 toxin detection utilizing a dual signal amplification strategy: Synergistic effects of bimetallic oxide (Ce-In)Ox and COFTAPB-DMTP. Sens. Actuators B Chem..

[118] Zhang S., Wang Y., Sheng Q., Yue T. (2023). Electrochemical aptasensor based on ZnO-Au nanocomposites for the determination of ochratoxin A in wine and beer. Processes.

[119] Evtugyn G., Porfireva A., Shamagsumova R., Hianik T. (2020). Advances in electrochemical aptasensors based on carbon nanomaterials. Chemosensors.

[120] Ayodele O.O., Adesina A.O., Pourianejad S., Averitt J., Ignatova T. (2021). Recent advances in nanomaterial-based aptasensors in medical diagnosis and therapy. Nanomaterials.

[121] Kaur H., Shorie M., Sharma M., Ganguli A.K., Sabherwal P. (2017). Bridged rebar graphene functionalized aptasensor for pathogenic *E. coli* O78:K80:H11 detection. Biosens. Bioelectron..

[122] Hasan M.R., Pulingam T., Appaturi J.N., Zifruddin A.N., Teh S.J., Lim T.W., Ibrahim F., Leo B.F., Thong K.L. (2018). Carbon nanotube-based aptasensor for sensitive electrochemical detection of whole-cell *Salmonella*. Anal. Biochem..

[123] Pathania P.K., Saini J.K., Vij S., Tewari R., Sabherwal P., Rishi P., Suri C.R. (2018). Aptamer functionalized MoS_2_-rGO nanocomposite based biosensor for the detection of Vi antigen. Biosens. Bioelectron..

[124] Li L., Yuan Y., Chen Y., Zhang P., Bai Y., Bai L. (2018). Aptamer based voltammetric biosensor for *Mycobacterium tuberculosis* antigen ESAT-6 using a nanohybrid material composed of reduced graphene oxide and a metal-organic framework. Microchim. Acta.

[125] Muniandy S., Dinshaw I.J., Teh S.J., Lai C.W., Ibrahim F., Thong K.L., Leo B.F. (2017). Graphene-based label-free electrochemical aptasensor for rapid and sensitive detection of foodborne pathogen. Anal. Bioanal. Chem..

[126] Dinshaw I.J., Muniandy S., Teh S.J., Ibrahim F., Leo B.F., Thong K.L. (2017). Development of an aptasensor using reduced graphene oxide chitosan complex to detect *Salmonella*. J. Electroanal. Chem..

[127] Muniandy S., Teh S.J., Appaturi J.N., Thong K.L., Lai C.W., Ibrahim F., Leo B.F. (2019). A reduced graphene oxide-titanium dioxide nanocomposite based electrochemical aptasensor for rapid and sensitive detection of *Salmonella enterica*. Bioelectrochemistry.

[128] Appaturi J.N., Pulingam T., Thong K.L., Muniandy S., Ahmad N., Leo B.F. (2020). Rapid and sensitive detection of *Salmonella* with reduced graphene oxide-carbon nanotube based electrochemical aptasensors. Anal. Biochem..

[129] Pourmadadi M., Shayeh J.S., Omidi M., Yazdian F., Alebouyeh M., Tayebi L. (2019). A glassy carbon electrode modified with reduced graphene oxide and gold nanoparticles for electrochemical aptasensing of lipopolysaccharides from *Escherichia coli* bacteria. Microchim. Acta.

[130] Zamani M., Pourmadadi M., Seyyed Ebrahimi S.A., Yazdian F., Shabani Shayeh J. (2022). A novel labeled and label-free dual electrochemical detection of endotoxin based on aptamer-conjugated magnetic reduced graphene oxide-gold nanocomposite. J. Electroanal. Chem..

[131] Mohsin D.H., Mashkour M.S., Fatemi F. (2021). Design of aptamer-based sensing platform using gold nanoparticles functionalized reduced graphene oxide for ultrasensitive detection of Hepatitis B virus. Chem. Pap..

[132] Ghanbari K., Roushani M. (2018). A nanohybrid probe based on double recognition of an aptamer MIP grafted onto a MWCNTs-Chit nanocomposite for sensing hepatitis C virus core antigen. Sens. Actuators B Chem..

[133] Rahmati Z., Roushani M., Hosseini H. (2021). Three-dimensional NiCo_2_O_4_ nanowires encapsulated in nitrogen-doped carbon networks as a high-performance aptamer stabilizer for impedimetric ultrasensitive detection of hepatitis C virus core antigen. Surf. Interf..

[134] Chekin F., Bagga K., Subramanian P., Jijie R., Singh S.K., Kurungot S., Boukherroub R., Szunerits S. (2018). Nucleic aptamer modified porous reduced graphene oxide/MoS_2_ based electrodes for viral detection: Application to human papillomavirus (HPV). Sens. Actuators B Chem..

[135] Bao W., Aodeng G., Ga L., Ai J. (2025). Aptamer-based electrochemical biosensors: Signal transduction mechanisms, application progress, and future trends. Sens. Actuator Rep..

[136] Abd-Ellatief R., Abd-Ellatief M.R. (2021). Electrochemical aptasensors: Current status and future perspectives. Diagnostics.

[137] Khan I., Saeed K., Khan I. (2019). Nanoparticles: Properties, applications and toxicities. Arab. J. Chem..

[138] Oberhaus F.V., Frense D., Beckmann D. (2020). Immobilization techniques for aptamers on gold electrodes for the electrochemical detection of proteins: A review. Biosensors.

[139] Carroll D.P., Mendes P.M. (2023). Recent advances in surface modification and antifouling strategies for electrochemical sensing in complex biofluids. Curr. Opin. Electrochem..

[140] Song Z., Han R., Yu K., Li R., Luo X. (2024). Antifouling strategies for electrochemical sensing in complex biological media. Microchim. Acta.

[141] Noguchi T., Nishitani S., Sakata T. (2024). Anti-biofouling effect of nanoporous gold electrode on nonspecific signal reduction for electrochemical biosensors. J. Electrochem. Soc..

[142] Jiang H., Sun Z., Guo Q., Weng X. (2021). Microfluidic thread-based electrochemical aptasensor for rapid detection of *Vibrio parahaemolyticus*. Biosens. Bioelectron..

